# High Prevalence of Vitamin D Deficiency in Pregnant Korean Women: The First Trimester and the Winter Season as Risk Factors for Vitamin D Deficiency

**DOI:** 10.3390/nu7053427

**Published:** 2015-05-11

**Authors:** Rihwa Choi, Seonwoo Kim, Heejin Yoo, Yoon Young Cho, Sun Wook Kim, Jae Hoon Chung, Soo-young Oh, Soo-Youn Lee

**Affiliations:** 1 Department of Laboratory Medicine and Genetics, Samsung Medical Center, Sungkyunkwan University School of Medicine, 81 Irwon-Ro, Gangnam-Gu, Seoul 135-710, Korea; E-Mail: rihwa.choi@samsung.com; 2 Biostatistics Team, Samsung Biomedical Research Institute, 81 Irwon-Ro, Gangnam-Gu, Seoul 135-710, Korea; E-Mail: seonwoo1.kim@samsung.com; 3 Biostatistics and Clinical Epidemiology Center, Samsung Medical Center, 81 Irwon-Ro, Gangnam-Gu, Seoul 135-710, Korea; E-Mail: heejin.yoo@sbri.co.kr; 4 Division of Endocrinology and Metabolism, Department of Medicine, Thyroid Center, Samsung Medical Center, Sungkyunkwan University School of Medicine, 81 Irwon-Ro, Gangnam-Gu, Seoul 135-710, Korea; E-Mails: yoonung2.cho@samsung.com (Y.Y.C.); sunwooksmc.kim@samsung.com (S.W.K.); jaeh.chung@samsung.com (J.H.C.); 5 Department of Obstetrics and Gynecology, Samsung Medical Center, Sungkyunkwan University School of Medicine, 81 Irwon-Ro, Gangnam-Gu, Seoul 135-710, Seoul, Korea

**Keywords:** vitamin D, pregnancy, mass spectrometry, Korea, nutrition

## Abstract

We investigated the vitamin D status of Korean women during pregnancy and assessed the effects of vitamin D deficiency on two pregnancy outcomes; preterm births and the births of small for gestational age. We measured the serum 25-hydroxyvitamin D levels in 220 pregnant Korean women who were recruited prospectively and compared these levels with those of 500 healthy non-pregnant women. We analyzed vitamin D status according to patient demographics, season, and obstetrical characteristics; moreover, we also assessed pregnancy outcomes. The overall prevalence of vitamin D deficiency(<20 ng/mL) in pregnant women and healthy non-pregnant women was 77.3% and 79.2%; respectively; and the prevalence of severe vitamin D deficiency (<10 ng/mL) was 28.6% and 7.2%; respectively (*p* < 0.05). Vitamin D deficiency was more prevalent in the winter (100%) than in the summer (45.5%) in pregnant Korean women. A higher risk of vitamin D deficiency was observed in the first trimester than in the third trimester (adjusted OR 4.3; *p* < 0.05). No significant association was observed between vitamin D deficiency and any of the pregnancy outcomes examined. Further research focusing on the long-term consequences of vitamin D deficiency during pregnancy in Korean women is warranted.

## 1. Introduction

Vitamin D status is a well-known determinant of skeletal health and influences the risk of fracture, rickets, osteomalacia, and osteoporosis [1]. Humans obtain vitamin D from exposure to sunlight and diet, which are the two main determinants of vitamin D status in a population. Vitamin D3 is formed by exposure of the skin to sunlight and can also be obtained from the diet via animal products, whereas vitamin D2 is obtained from the diet via plant sources [1]. Vitamin D (hereafter used to refer to both D2 and D3) from the skin and diet is converted into the circulating metabolite 25-hydroxyvitamin D—25(OH)D, including both 25(OH)D2 and 25(OH)D3—in the liver. This metabolite is often used as a biomarker to determine a patient’s vitamin D status [2]. The 25(OH)D is metabolized in the kidneys into its active form, 1,25-dihydroxyvitamin D—1,25(OH)2D. The active form circulates in the blood at a significantly lower concentration (approximately 1/1000) compared with the concentration of 25(OH)D [1]. Circulating vitamin D, 25(OH)D, and 1,25(OH)2D are all bound to vitamin D-binding protein, a specific transporter protein. In target tissues, 1,25(OH)2D exerts its actions by associating with the VDR nuclear receptor (vitamin D receptor). Since the VDR is ubiquitously expressed in most cell types, including brain, prostate, breast, placenta, and immune, vitamin D has been hypothesized to have a number of functions outside the skeletal system [1].

Due to the importance of vitamin D, many concerns have been raised regarding the functional impacts of maternal vitamin D deficiency on multiple adverse health outcomes in mothers and their offspring. Moreover, low maternal levels of 25-hydroxyvitamin D have been suggested to be associated with a number of adverse obstetrical and neonatal outcomes [3,4]. Vitamin deficiency is a modifiable factor; therefore, it is important to determine the optimal vitamin D status during pregnancy. In the context of increasing clinical concern regarding the high prevalence of vitamin D deficiency worldwide, different prevalences of vitamin D deficiency have been reported in different geographic regions and latitudes. These prevalences have been determined by different diagnostic methods [5,6].

Multiple studies have shown that immunoassays may be limited by the cross-reactivity of antibodies and by non-equimolar recognition of the D2 and D3 forms of 25(OH)D, thereby overestimating the serum 25(OH)D concentration and the influence of vitamin D binding protein, which is known to circulate at higher concentrations in pregnant women [7,8]. Therefore, liquid chromatography-tandem mass spectrometry (LC-MS/MS) has been used as a reference method to accurately estimate patient vitamin D status [9,10].

However, a large population-based estimate of the vitamin D status of pregnant Korean women has not yet been performed. Therefore, this study aimed to investigate the vitamin D status of pregnant Korean women. This study also set out to investigate the determinants of vitamin D status during pregnancy and to assess the impact of vitamin D deficiency on pregnancy outcomes.

## 2. Methods

### 2.1. Ethics Statement

This study was conducted according to the guidelines laid down in the Declaration of Helsinki, and all procedures involving human subjects were approved by the Institutional Review Board of Samsung Medical Center (2011-12 & SMC 2011-12-041-001). All subjects provided written consent for their participation in this study.

### 2.2. Study Population

The target population of this study comprised pregnant women living in an urban area of South Korea (latitude 36°N) in any trimester of pregnancy. During the period of April 2012–September 2013, 282 pregnant women were prospectively recruited and followed. Among these women, 62 were excluded for one of the following reasons: Lack of information about basal characteristics such as prepregnancy body mass index (BMI), smoking, alcohol consumption, and occupation (*n* = 5), history of concurrent serious medical disease that could affect pregnancy outcomes (*n* = 5), history of intra-abdominal surgery (*n* = 11), history of surgery of the uterine cervix (*n* = 24) [11], and a lack of information regarding pregnancy outcomes due to follow-up loss (*n* = 17). As a result, 220 women were ultimately enrolled in the study. The vitamin D levels in these pregnant women were compared with those from healthy nonpregnant women of childbearing age (*n* = 500, 24–44 years) who visited a health-promotion center at Samsung Medical Center during the study period.

### 2.3. Data Collection

Blood samples were collected from the antecubital vein from pregnant women at the first prenatal consultation in any trimester of their pregnancy. Information about demographic characteristics, sociodemographic characteristics, smoking status, alcohol consumption during pregnancy and during the four weeks prior to the last menstrual period, diseases, medications, and obstetrical and gynecological history—*i.e.*, parity (number of deliveries) and gravity (number of pregnancies)—was gathered by obstetrical nurses via questionnaires at the initial prenatal consultation. Some information was also obtained from electronic medical records. For all women included in the study, the prepregnant BMI was obtained from the self-reported weight and height recorded by the gynecologist during the prenatal consultation. BMIs were classified according to published cutoffs for Asian populations [12]. If the prepregnant body weight was not known, the first trimester BMI was used as a proxy for prepregnancy BMI for all women in their first, second, and third trimesters. Gestational age was determined according to the last menstrual period (LMP) and ultrasonography results. For the approximate 12% of all ultrasound gestational age estimates that differed by >10 days from LMP pregnancy dating, ultrasound dating was used. Neonatal outcomes were obtained from hospital medical records.

### 2.4. Definitions of Adverse Pregnancy Outcomes

A preterm birth was defined as a birth at less than 37 weeks of gestation. Neonates with birth weights below the 10th percentile for their gestational age as determined by birth weight percentile nomograms (National Data from Korean Health Insurance Review & Assessment Service 2009) were considered to be small for gestational age (SGA).

### 2.5. Laboratory Analyses

Approximately 500 μL serum was separated from 2 mL whole blood collected by venipuncture in a plain tube. Serum 25(OH)D2 and 25(OH)D3 concentrations were measured by LC-MS/MS with an Agilent 1200 LC 2D system connected to an Agilent 6460 Triple Quad MS (Agilent Technologies, Waldbronn, Germany). Vitamin D levels were detected in positive mode using the multiple reaction monitoring technique. The total 25(OH)D concentration was calculated as the sum of the serum 25(OH)D2 and 25(OH)D3 concentrations. The inter-assay and intra-assay coefficients of variation for this method were 5.5% and 7.1%, respectively. Based on published definitions of vitamin D status [1], we categorized 25(OH)D ≥30 ng/mL (≥75 nmol/L) as sufficient and 25(OH)D 20–29.9 ng/mL(50–74.9 nmol/L) as suboptimal. Vitamin D deficiency was defined as 25(OH)D <20 ng/mL (<50 nmol/L) [13] and severe vitamin D deficiency was defined as 25(OH)D <10 ng/mL (<25 nmol/L) [14]. Since the optimal vitamin D concentration in pregnancy has been debated by groups such as the Institute of Medicine (IOM) and the Endocrine Society, no gold standard measurement method has been used to gather data regarding vitamin D status in large samples of Korean women, and little data are available regarding vitamin D status and adverse birth outcomes, the Endocrine Society’s criteria for vitamin D status [1] were applied to assess the overall vitamin D status of pregnant women. The IOM’s cutoff for vitamin D deficiency (<20 ng/mL) [13] was employed to analyze the association of vitamin D deficiency with adverse pregnancy and neonatal outcomes.

### 2.6. Statistical Analysis

Characteristics are presented as frequencies and percentages. Since age, prepregnancy BMI, and serum 25(OH)D levels were not normally distributed, nonparametric methods were used. The median was used as the measure of central tendency. Differences between trimesters, seasons of blood draw and of 25(OH)D measurements, and age groups were explored using the Kruskal-Wallis equality-of-populations rank test.

The odds of having a vitamin D deficiency (serum 25(OH)D < 20 ng/mL) *versus* a nondeficient vitamin D status were estimated through multivariable-adjusted logistic regression models. The following variables were entered as predictors in the model: Age, trimester, seasons of blood draw and of 25(OH)D measurements, education level, job, type of current pregnancy, concurrent pregnancy status, gravity, parity, previous or concurrent medical history (with the exception of intra-abdominal surgery), and gynecological disease history (with the exception of uterine cervix disease). The appropriateness of the sample size was validated by calculating the width of the confidence interval (CI). At the expected vitamin D deficiency rate of 80%, the 95% CI for vitamin D levels was calculated to be ±10%. The precisions of these two estimates were sufficient; thus, the size of this study was adequate for statistical analysis. A *p* value <0.05 was considered statistically significant. All *p* values were corrected by Bonferroni’s method for multiple testing.

## 3. Results

### 3.1. General Characteristics

In total, 220 pregnant women in Korea participated in this study. The median age was 32.0 years old. Only 24 women (10.9%) had a prepregnancy BMI above 24 and no participant had a history of drinking alcohol during pregnancy. Only one participant had a history of smoking during pregnancy. More than three-quarters of all participants had more than 12 years of education, and over two-thirds had indoor jobs. The basal characteristics of the study population are summarized in Table 1.

### 3.2. Serum 25(OH)D Levels in Pregnant Korean Women

**Figure 1:**
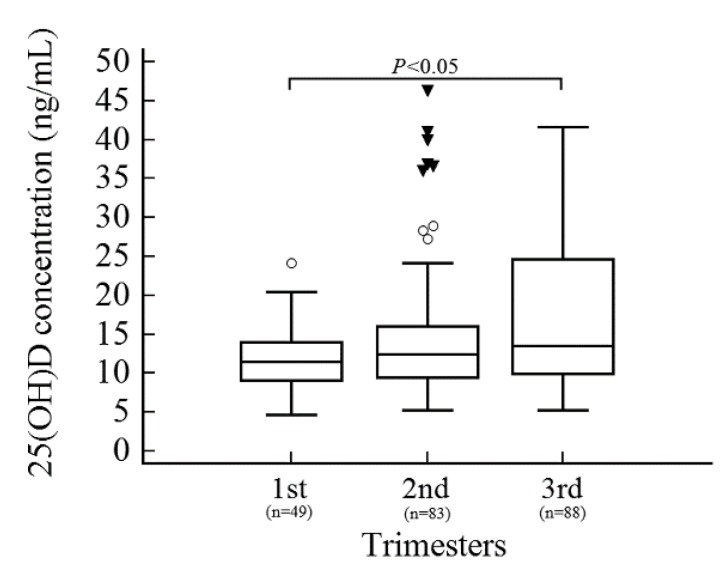
Serum 25-hydroxyvitamin D—25(OH)D—concentration according to trimester. Of particular note, the 25(OH)D concentration was significantly higher in the third trimester compared with the first trimester (*p* < 0.05).

The median serum 25(OH)D concentration of all participants (*n* = 220) was 12.6 ng/mL. Serum 25(OH)D concentrations during the three trimesters are shown in Table 2 and Figure 1. According to pooled analysis, vitamin D concentrations differed significantly only between the first and third trimesters: 11.5 ng/mL during the first trimester *versus* 13.6 ng/mL during the third trimester (*p* < 0.05). Pooled analysis also revealed that the median serum 25(OH)D concentrations were10.8 ng/mL in the spring, 20.5 ng/mL in the summer, 13.9 ng/mL in the fall, and 9.4 ng/mL in the winter. The serum 25(OH)D concentrations were significantly different between the spring and summer, the spring and fall, the summer and winter, and the fall and winter (*p* < 0.05). However, the serum 25(OH)D concentrations were not significantly different between the winter and spring or between the summer and fall (*p* > 0.05). Although no women in their third trimester of pregnancy were tested during the winter, peaks of 25(OH)D concentration were observed in the summer for all women who were tested in their first and third trimesters.

### 3.3. Prevalences of Vitamin D Deficiency and Insufficiency in Pregnant Women

The percentages of vitamin D deficiency and insufficiency are shown in Table 2. A high prevalence of vitamin D deficiency was observed in pregnant women during all trimesters and also in healthy nonpregnant women. The overall prevalence of vitamin D deficiency—25(OH)D <20 ng/mL—in pregnant women was 77.3%; moreover, only 19 women (8.6%) had a serum 25(OH)D concentration >30 ng/mL, which is considered the optimal level. The median 25(OH)D concentration was higher in healthy nonpregnant women (15.4 ng/mL) compared with pregnant women (12.6 ng/mL). In contrast to pregnant women, among whom the prevalence of severe vitamin D deficiency was 28.6%, the prevalence of severe vitamin D deficiency among healthy nonpregnant women was 7.2%.

**Table 1:** Basic parameters of pregnant Korean women (*n* = 220).

Parameter	Total (*n* = 220)		First Trimester (*n* = 49)		Second Trimester (*n* = 83)		Third Trimester (*n* = 88)		*p*
	Median	Range	Median	Range	Median	Range	Median	Range	
**Age, years**	32.0	24.0–43.9	31.0	24.0–41.3	32.0	26.0–43.9	32.0	25.0–39.0	0.72
**Prepregnant BMI (kg/m^2^)**	20.2	16.0–28.5	20.3	16.0–26.3	20.8	16.3–28.5	19.8	16.0–27.5	0.21
Prepregnant BMI *	*n*	%	*n*	%	*n*	%	*n*	%	0.84
Underweight (BMI < 18.0)	27	12.3	6	12.2	10	10.0	11	12.5	
Healthy normal (BMI 18.0–23.9)	169	76.8	38	77.6	63	75.9	68	77.3	
Overweight (BMI 24.0–26.9)	20	9.1	5	10.2	7	8.4	8	9.1	
Obese (BMI ≥ 27.0)	4	1.8	0	0.0	3	3.6	1	1.2	
**Season** ^†^									0.10
Spring	98	44.5	20	40.8	35	42.2	43	48.9	
Summer	22	10.0	5	10.2	7	8.4	10	11.4	
Fall	87	39.5	18	39.7	34	40.9	35	39.8	
Winter	13	5.9	6	12.2	7	8.4	0	0.0	
**Education level** ^‡^									0.53
Low	12	5.5	3	6.1	6	7.2	3	3.4	
High	208	94.5	46	93.9	77	92.8	85	96.6	
**Job**									0.66
Any job	70	31.8	35	71.4	58	69.9	57	64.8	
Homemaker	150	68.2	14	28.6	25	30.1	31	35.2	
**Type of current pregnancy**									0.56
Spontaneous pregnancy	213	96.8	48	98.0	79	95.2	86	97.7	
Artificial pregnancy	7	3.2	1	2.0	4	4.8	2	2.3	
**Single or multiple pregnancy**									0.90
Singleton	217	98.6	48	98.0	82	98.8	87	98.9	
Twins	3	1.4	1	2.0	1	1.2	1	1.1	
**Gravida**									0.57
Primigravida	142	64.5	29	59.2	53	63.9	60	68.2	
Multigravida	78	35.5	20	40.8	30	36.1	28	31.8	
	*n*	%	*n*	%	*n*	%	*n*	%	
**Parity**									0.36
0 (nullipara)	136	61.8	27	55.1	50	60.2	59	67.0	
≥1	84	38.2	22	44.9	33	39.8	29	33.0	
**Previous or concurrent medical history**									0.30
Yes	68	30.9	17	34.7	29	34.9	22	25.0	
No	152	69.1	32	65.3	54	65.1	66	75.0	
**Gynecological disease history**									0.88
Yes	44	20.0	9	18.4	16	19.3	19	21.6	
No	176	80.0	40	81.6	67	80.7	69	78.4	

BMI: Body mass index; *: BMI classification for Asian populations was performed as described in [12]; **^†^**: Season of blood draw and of 25(OH)D measurements; **^‡^**: Women who were educated ≤12 years were categorized as *low* and >12 years were categorized as *high*.

**Table 2:** The 25-hydroxyvitamin D—25(OH)D—concentrations in 220 pregnant Korean women across three trimesters and in 500 healthy nonpregnant women.

25(OH)D (ng/mL)	Healthy Nonpregnant Women * (*n* = 500)	All Pregnant Women (*n* = 220)	First Trimester (*n* = 49)	Second Trimester (*n* = 83)	Third Trimester (*n* = 88)
Median ^†,^^‡^	15.4	12.6	11.5	12.5	13.6
IQR	12.7–19.7	9.7–17.3	9.1–14.0	9.5–16.0	9.9–24.7
95% CI	14.8–15.8	11.9–13.3	10.0–13.0	11.1–13.4	12.1–16.6
Range	5.8–40.4	4.7–46.3	4.7–24.2	5.3–46.3	5.3–41.6
% <10 ng/mL ^†,§,¶,^^||^	7.2	28.6	32.7	28.9	26.1
% <20 ng/mL ^§^	79.2	77.3	91.8	80.7	65.9
% <30 ng/mL ^†,§^	98.0	91.4	100.0	92.8	85.2

IQR: Interquartile range; *: Data were obtained from healthy nonpregnant women of childbearing age (24–44 years old) who visited a health-promotion center at Samsung Medical Center and volunteered to give blood during the study period; ^†^: The serum 25(OH)D concentration, prevalence of vitamin D deficiency, and prevalence of women with suboptimal 25(OH)D levels (<30 ng/mL) were all significantly different between healthy nonpregnant women and pregnant women (*p* < 0.05); ^‡^: The serum 25(OH)D concentration was significantly different between healthy nonpregnant women and pregnant women at each trimester (*p* < 0.05), except for pregnant women in their third trimester, after *post-hoc* analysis; §: The prevalences of severe vitamin D deficiency—25(OH)D <10 ng/mL, vitamin D deficiency—25(OH)D <20 ng/mL—and suboptimal 25(OH)D levels (<30 ng/mL) were significantly different among healthy nonpregnant women and pregnant women at each trimester; ^¶^: A higher prevalence of severe vitamin D deficiency—25(OH)D <10 ng/mL—was observed in pregnant women than in healthy nonpregnant women; ^||^: The prevalences of severe vitamin D deficiency in the three trimesters were not significantly different among pregnant women (*p* > 0.05).

**Figure 2:**
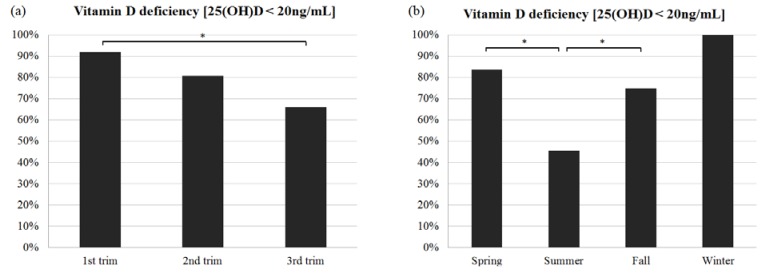
Prevalence of vitamin D deficiency (25(OH)D < 20 ng/mL) according to trimester and season. (**a**) Prevalence of vitamin D deficiency by trimester. The prevalence of vitamin D deficiency was significantly higher in the first trimester compared with the third trimester (*p* < 0.05); (**b**) Prevalence of vitamin D deficiency according to season of blood draw and 25(OH)D measurements. The prevalence of vitamin D deficiency was lower in the summer than in the spring or fall (*p* < 0.05); *: Statistically significant differences according to multivariable analysis (*p* < 0.05).

The percentage of vitamin D deficiency was higher in the first trimester (91.8%) than in the third trimester (65.9%) and was also higher in the winter (100.0%) than in the summer (45.5%) (*p* < 0.05). Interestingly, the prevalence of vitamin D deficiency decreased as pregnancy progressed: 91.8% during the first trimester, 80.7% during the second trimester, and 77.3% during the third trimester. All participants had vitamin D insufficiency in the first trimester. Pooled analyses of all blood samples revealed significant differences in the prevalences of vitamin D deficiency and the 25(OH)D concentrations across seasons. The prevalences of vitamin D deficiency were 84.7%, 45.5%, 73.7%, and 100% in the spring, summer, fall, and winter, respectively, with a significantly lower prevalence in the summer compared with the spring and fall (*p* < 0.05). The difference in the prevalence of vitamin D deficiency between the summer and the winter was not significant, probably because all women who were tested in the winter had a vitamin D deficiency and only 13 pregnant women were included during the winter. Thus, this statistical analysis was likely influenced by the small sample size (Figure 2). Additional analyses for potential interactions between season and trimester were performed for further statistical modeling. However, the effect of the first trimester occurring during winter was not significantly different than other season-trimester combinations (*p* = 0.4983). This finding could be due to the small sample size during winter (*i.e.*, no pregnant women in their third trimester were included in the winter in this study population). Thus, although an interaction term was included in an earlier iteration of the statistical model, it was not significant and thus, it was not included in the final model.

**Table 3:** Maternal characteristics and vitamin D status of 220 pregnant Korean women.

Vitamin D Cutoff	Total		Deficiency		Suboptimal		Optimal		*p*
			<20 ng/mL		20–29 ng/mL		30–100 ng/mL		
	*n*	%	*n*	%	*n*	%	*n*	%	
**All women**	220	100.0	170	77.3	31	14.1	19	8.6
**Prepregnant BMI** ^*^									>1
Underweight (BMI < 18.0)	27	12.3	21	77.8	5	18.5	1	3.7
Healthy normal (BMI 18.0–23.9)	169	76.8	129	76.3	25	14.8	15	8.9
Overweight (BMI 24.0–26.9)	20	9.1	17	85.0	1	5.0	2	10.0
Obese (BMI ≥ 27.0)	4	1.9	3	75.0	0	0.0	1	25.0
**Age**									0.53
<30 years	56	25.5	46	82.1	8	14.3	2	3.6
30–35 years	119	54.1	86	72.3	19	16.0	14	11.8
35–40 years	45	20.5	38	84.4	4	8.9	3	6.7
**Trimester**									0.012
First trimester	49	22.3	45	91.8	4	8.2	0	0.0
Second trimester	83	37.7	67	80.7	10	12.0	6	7.2
Third trimester	88	40.0	58	65.9	17	19.3	13	14.8
**Season** ^†^									0.007
Spring	98	44.5	82	83.7	9	9.2	7	7.1
Summer	22	10.0	10	45.5	8	36.4	4	18.2
Fall	87	39.5	65	74.7	14	16.1	8	9.2
Winter	13	5.9	13	100.0	0	0.0	0	0.0
**Education level**									0.84
≤12 years	12	5.5	11	91.7	1	8.3	0	0.0
>12 years	208	94.5	159	76.4	30	14.4	19	9.1
**Job**									0.61
Any job	150	68.2	119	79.3	21	14.0	10	6.7
Homemaker	70	31.8	51	72.9	10	14.3	9	12.9
**Type of current pregnancy**									0.13
Spontaneous	213	96.8	167	78.4	29	13.6	17	8.0
Artificial insemination	7	3.2	3	42.9	2	28.6	2	28.6
**Single or multiple pregnancy**									0.31
Singleton	217	98.6	169	77.9	30	13.8	18	8.3
Twins	3	1.4	1	33.3	1	33.3	1	33.3
**Gravity**									>1
Primigravida	142	64.5	107	75.4	21	14.8	14	9.9
Multigravida	78	35.5	63	80.8	10	12.8	5	6.4
**Parity**									0.96
0 (nullipara)	136	61.8	102	75.0	20	14.7	14	10.3
≥1	84	38.2	68	81.0	11	13.1	5	6.0
**Previous or concurrent medical history**									0.69
Yes	152	69.1	120	78.9	18	11.8	14	9.2
No	68	30.9	50	73.5	13	19.1	5	7.4
**Gynecological disease history**									0.18
Yes	176	80.0	131	74.4	29	16.5	16	9.1
No	44	20.0	39	88.6	2	4.5	3	6.8

*: BMI classification for Asian populations was performed as described in [12]. **^†^**: Season of blood draw and of 25(OH)D measurements.

**Table 4:** Risk of vitamin D deficiency during pregnancy in 220 Korean women.

Risk of Vitamin D Deficiency	Number of Subjects	VitD-Deficient Subjects (<20 ng/mL)		Unadjusted Odds Ratio	95% CI		*p*	Adjusted Odds Ratio	95% CI		*p*
		*n*	%		Lower	Upper			Lower	Upper	
**Age**							0.16				0.27
<30 years	56	46	82.1	Reference				Reference			
30–35 years	119	86	72.3	0.567	0.229	1.403		0.523	0.186	1.475	
35–40 years	45	38	84.4	1.180	0.352	3.953		0.926	0.242	3.553	
**Prepregnant BMI** *							0.86				0.74
Underweight (BMI < 18.0)	27	21	77.8	1.085	0.330	3.567		0.628	0.168	2.345	
Healthy normal (BMI 18.0–23.9)	169	129	76.3	Reference				Reference			
Overweight (BMI 24.0–26.9)	20	17	85.0	1.757	0.369	8.3670		0.789	0.148	4.217	
Obese (BMI ≥ 27.0)	4	3	75.0	0.930	0.058	15.271		0.326	0.010	11.007	
**Trimester**							0.003				0.023
First trimester	49	45	91.8	5.819	1.629	20.792		4.274	1.205	15.159	
Second trimester	83	67	80.7	2.166	0.971	4.830		2.013	0.818	4.957	
Third trimester	88	58	65.9	Reference				Reference			
**Season** ^†^							0.002				0.003
Spring	98	82	83.7	5.952	1.770	20.018		8.026	1.973	32.650	
Summer	22	10	45.5	Reference				Reference			
Fall	87	65	74.7	3.465	1.063	11.296		4.346	1.113	16.970	
Winter	13	13	100.0	32.157	0.777	1330.6	26.322	0.596	1161.7	
**Education level**							0.25				0.40
≤12 years	12	11	91.7	Reference				Reference			
>12 years	208	159	76.4	0.295	0.037	2.343		0.446	0.067	2.969	
**Job**							0.29				0.10
Any job	150	119	79.3	1.430	0.740	2.763		1.932	0.880	4.242	
Homemaker	70	51	72.9	Reference				Reference			
**Type of current pregnancy**							0.044				0.11
Spontaneous	213	167	78.4	Reference				Reference			
Artificial insemination	7	3	42.9	0.207	0.045	0.956		0.127	0.010	1.610	
**Single or multiple pregnancy**							0.11				0.78
Singleton	217	169	77.9	Reference				Reference			
Twins	3	1	33.3	0.142	0.013	1.601		0.559	0.009	35.596	
Gravity ^‡^							0.72				
Primigravida	142	107	75.4	Reference							
Multigravida	78	63	80.8	1.374	0.631	2.991					
**Parity**							0.62				0.27
0 (nullipara)	136	102	75.0	Reference				Reference			
≥1	84	68	81.0	1.417	0.659	3.0436		1.586	0.702	3.580	
**Previous or concurrent medical history**							0.38				0.36
Yes	152	120	78.9	0.741	0.381	1.4400		0.698	0.324	1.503	
No	68	50	73.5	Reference				Reference			
**Gynecological disease history**							0.05				0.027
Yes	176	131	74.4	2.679	0.995	7.2150		3.466	1.155	10.399	
No	44	39	88.6	Reference				Reference			

*: BMI classification for Asian populations was performed as described in [12]; **^†^**: Season of blood draw and of 25(OH)D measurements; ^‡^: The adjusted odds ratio for multivariate analysis was not calculated due to multicolinearity between gravity and parity.

### 3.4. Factors Associated with Vitamin D Deficiency during Pregnancy

Maternal characteristics, stratified by vitamin D status, are shown in Table 3. Age, education level, occupation, type of current pregnancy, number of concurrent pregnancies, gravity, parity, previous/concurrent medical history (with the exception of intra-abdominal surgery), and gynecological disease history (with the exception of uterine cervix disease) were not significantly different among the three groups of pregnant women as stratified by vitamin D status.

Factors associated with vitamin D deficiency over the course of pregnancy are shown in Table 4. Multiple logistic regression models for identifying independent predictors of vitamin D deficiency revealed that the winter season and the first trimester were independent predictors of vitamin D deficiency. The risk of vitamin D deficiency was significantly higher in the first *vs.* the third trimester (adjusted OR 4.2744; *p* < 0.05) and in the spring (adjusted OR 8.0258; *p* < 0.05). Artificial insemination pregnancies had a lower risk of vitamin D deficiency than spontaneous pregnancies according to univariate analysis, but this association was not seen in multivariate analysis. No significant associations were observed between any other factors and vitamin D deficiency.

### 3.5. Associations between Vitamin D Deficiency and Pregnancy Outcomes

The associations between adverse pregnancy outcomes and vitamin D deficiency (<20 ng/mL) during pregnancy were analyzed (Table 5). Among the 220 pregnant women, 54 (24.5%) had adverse pregnancy outcomes. Specifically, nine delivered preterm babies (4.1%) and 24 had babies small for their gestational age (10.9%). The prevalences of vitamin D deficiency were 77.8% (7/9) among the women who delivered preterm babies and 62.5% (15/24) among the women who had babies small for their gestational age.

No significant associations were observed between vitamin D deficiency and preterm delivery or SGA babies according to univariate or multivariate logistic regression analyses.

**Table 5:** Associations between vitamin D deficiency, preterm babies, and SGA babies.

Outcomes	Preterm			SGA		
	Preterm (−)	Preterm (+)	*p*	SGA (−)	SGA (+)	*p*
Number of subjects	211	9		196	24	
Number with VitD deficiency *	163	7		155	15	
% with VitD deficiency *	77.3	77.8		79.1	62.5	
Unadjusted OR (95% CI)	Reference	1.030 (0.207–5.124)	0.97	Reference	0.441 (0.180–1.079)	0.07
Adjusted OR (95% CI)	Reference	0.699 (0.144–3.402)	0.66	Reference	0.448 (0.149–1.351)	0.15

SGA: Small for gestational age; OR: Odds ratio; *: 25(OH)D < 20 ng/mL.

## 4. Discussion

The strengths of this study include its prospective study design, the fairly ethnically homogenous sample of adult Koreans, and the use of the gold standard LC-MS/MS method to measure 25(OH)D concentrations. Also, to the best of our knowledge, this is the first report of vitamin D status in Korean pregnant women, the first risk assessment for vitamin D deficiency during pregnancy, and the first investigation of the effect of vitamin D deficiency on pregnancy outcomes.

This study showed a high prevalence (77.3%) of vitamin D deficiency during pregnancy. Although many reports have reported high prevalences of vitamin D deficiency among pregnant women, most of these studies have focused on white and black pregnant women. Only a few studies have assessed vitamin D status in pregnant women living in Asia [15,16,17,18,19]. Sunlight exposure at different latitudes is likely to be an important factor that influences vitamin D status. The results of previous studies of vitamin D status among pregnant women in Asia at variable latitudes and in other regions near 36°N [3,4,20,21,22,23,24], which is similar to the latitude of the present study, are summarized in Table 6. The high prevalence of vitamin D deficiency observed in the present study is comparable with the findings of previous studies of Asian populations [15,16,17,18,19]. These studies tested vitamin D status at different gestational periods and used different cutoffs to define vitamin D deficiency. Moreover, most studies measured vitamin D levels by immunoassays rather than LC-MS/MS, thereby hindering direct comparisons of reported values of 25(OH)D concentrations. A recent study of 311 pregnant Chinese women in Guiyang, China reported a slightly higher prevalence of vitamin D deficiency (83.6%) with a slightly higher mean 25(OH)D concentration (14.69 ng/mL) [19]. This study used LC-MS/MS for measurement and sampled during the second and third trimesters. Additional studies using accurate measurement methods are needed to obtain more robust estimates of vitamin D status among Asian populations. In the present study, we found that the median 25(OH)D level among pregnant Korean women was significantly lower in the winter (9.4 ng/mL) than in the summer (20.5 ng/mL) or the fall (13.9 ng/mL) (*p* < 0.05). Consistent with this finding, the prevalence of vitamin D deficiency was much higher in the winter (100%) than in the summer (45.5%) (*p* < 0.05). Even in the summer, a vitamin D deficiency was still found in 45.5% of all women in our cohort. These results are comparable with those of previous studies in China, Greece, Iran, the Spanish Mediterranean seacoast, and California (USA) [15,19,22,23]. One previous study in Japan found no significant seasonal variation of vitamin D levels in pregnant women [16], although the highest concentration occurred in the fall. Moreover, a high prevalence of vitamin D deficiency was seen in all four seasons. Thus, it appears to be a general trend that the vitamin D levels in Asian populations are higher in the summer than in the winter.

**Table 6:** Serum 25(OH)D concentrations in pregnant women in Asian populations and in regions at latitudes near 36°N.

Ref.	Region	Lat. (°N) *	*N* of Preg	GA at Blood Sampling	25(OH)D Concentration				% <20 ng/mL ^§^ (% <50 nmol/L)	Pregnancy and Birth Outcome	Significant Association (*p* < 0.05)	Method	
					Presented as	Reported Value	Units ^†^	Converted to ng/mL ^‡^				
**Asia**												
This study	South Korea	36	220	First, Second, Third trimesters	median (IQR)	12.6 (9.65–17.30)	ng/mL	12.6	77.3%	PROM, preterm delivery, SGA	No	LC-MS/MS
[16]	Tokai, Japan	35.3	93 ^¶^	30 weeks	mean ± SD	14.5 ± 5.0	ng/mL	14.5	89.5%	premature delivery	premature delivery	RIA
[17]	Beijing, China **	39.9	125	15–20 weeks	mean ± SD	28.4 ± 9.5	nmol/L	11.42	96.8%	NA		ELISA
[19]	Guiyang, China	NA *	311	Second and third trimesters	mean ± SD	14.69 ± 6.81	ng/mL	14.69	83.6%	NA		LC-MS/MS
[17]	Beijing, China	39.9	70	Prior to labor	mean ± SE	28.64 ± 1.41	nmol/L	11.47	90.2%	birth weight, birth length, HC	birth weight, birth length	ELISA
[15].	Nanjing, China	31	152	24–28 weeks	mean ± SD	10.9 ± 4.78	ng/mL	10.9	in winter 96.1% in summer 94.7%	NA		ELISA
[18]	Chengdu, China	30.7	77	Before labor	mean ± SD	35.95 ± 19.7	nmol/L	14.40	NA	NA		EIA
**Studies at regions near 36°N**												
[21]	Tehran, Iran **	NA *	552	Delivery	mean ± 2 SD	27.8 ± 21.71	nmol/L	11.1	NA	birth height, weight, HC, post. & ant. fontanel diameter, Apgar score	No	RIA
[20]	USA	NA *	928	First, second, third trimesters	mean (95% CI)	65 (61–68)	nmol/L	26.0	33.8%	NA		RIA
[23]	Almeria, Spain	36	502	11–14 weeks	median (IQR)	27.4 (20.9–32.8)	ng/mL	27.4	22.7%	NA		ECLIA
[3]	Almeria, Spain ^‡‡^	36	466	First, third trimesters	NA	NA	ng/mL	NA	23.4%	^§§^ PROM, preterm delivery, SGA, etc.	No	ECLIA
[22]	Athens, Greece	NA *	123	Delivery	median (IQR)	16.4 (11–21.1)	ng/mL	16.4	NA	NA		CLIA
[4]	Izmir, Turkey	38.25	300	≥37 weeks	mean ± SD	11.5 ± 5.4	ng/mL	11.5	90.3%	NA		CLIA
[24]	Ankara, Turkey **	40	79	Third trimester	mean ± SD	11.95 + 7.20	ng/mL	11.95	NA	birth height, weight, HC, post. & ant. fontanel diameter, MUAC, Apgar score	No	HPLC

Ref.: Reference; Lat.: Latitude; *N* of preg.: Number of enrolled pregnant women; GA: Gestational age; IQR: Interquartile range; PROM: Premature rupture of membranes; SGA: Small for gestational age; LC-MS/MS: Liquid chromatography-tandem mass spectrometry; EIA: Enzyme immunoassay; RIA: Radioimmunoassay; ELISA: Enzyme linked immunosorbent assay; ECLIA: Electrochemiluminescence assay; CLIA: Chemiluminescence immunoassay; early, first measurement (early stage of pregnancy); late, second measurement (late stage of pregnancy); NA: Not available; HC: Head circumference; MUAC: Mid-upper arm circumference; *: Latitude information was obtained from maps, but was not reported in the referenced articles themselves; **^†^**: Reported units for 25(OH)D concentration in the referenced articles; **^‡^**: To convert the 25(OH)D values to nanomoles per liter, the values were multiplied by 2.496 (1 ng/mL is equivalent to 2.496 nmol/L). Only median or mean values were included in the table; **^§^**: A 25(OH)D concentration <20 ng/mL (<50 nmol/L) was defined as vitamin D deficiency; ^¶^: Including 14 cases with threatened premature delivery; **: Sampled only in winter; ^‡‡:^ This study was the second phase of a study performed using a subset of participants recruited in a study by Perez-Lopez *et al.* [23]; ^§§^: Obstetric and neonatal outcomes included labor initiation, route of delivery, PROM, hypertensive state, presence of gestational diabetes, intrauterine fetal demise, preterm birth, neonatal gender, Apgar score at birth, SGA, and congenital malformation.

In the present study, analysis of vitamin D status according to trimester revealed that being in the first trimester was a risk factor for vitamin D deficiency in pregnant Korean women. During pregnancy, the serum levels of 1,25(OH)D increase up to 2-fold starting at 10–12 weeks of gestation and reaching a maximum in the third trimester [25]. However, it is unclear whether 25(OH)D levels steadily increase throughout pregnancy [20]. The lower vitamin D concentration in the first trimester observed in the present study is comparable with previous studies in Thailand [26] and the United States [20], but conflicts with a study in Delhi, India, which found no significant difference among trimesters [27]. However, the latitude of Delhi is 28.6°N, and this region enjoys abundant sunlight during most of the year, in contrast to the region of the present study (36°N). This study is the first to assess vitamin D status across pregnancy trimesters in Asia at latitudes near 36°N. Although the National Health and Nutrition Examination Survey was conducted in the United States at a latitude similar to this study (36°N) [20] and reported that later trimester was independently associated with a higher 25(OH)D level—Asian pregnant women were only a small percentage of the participants and were categorized with other ethnic minorities. It is of particular note that, although the latitudes of the studied regions were similar to the latitude in this study, the vitamin D levels were higher in Western countries (the United States and the Spanish Mediterranean seacoast) than in Asian countries, including Korea. This difference could be due to other covariates such as demographics, genetic backgrounds of different ethnic groups, vitamin D supplement use, and outdoor activities [1,14]. Our results are most relevant to vitamin D studies of Asian populations in temperate climate areas.

In the present study, we compared the prevalence of vitamin D deficiency between pregnant women and healthy nonpregnant women. A high prevalence was observed in both groups (79.2% in healthy nonpregnant women and 77.3% in pregnant women). The finding that severe vitamin D deficiency was more prevalent in pregnant women (28.6%) than in nonpregnant women (7.2%) suggests that pregnancy itself could be a risk factor for vitamin D deficiency. This finding could be due to physiological changes resulting from nutrient demand and loss during pregnancy [14].

Interestingly, in addition to trimester and season as risk factors for vitamin D deficiency, we also identified a history of gynecological disease (*i.e.*, leiomyomas of the uterus or benign ovarian cysts) as a risk factor for vitamin D deficiency through multivariable logistic regression analysis (adjusted OR 3.4662; 95% CI 1.1550–10.3999; *p* < 0.05). Previous studies have suggested an association between vitamin D status and uterine diseases such as uterine myoma and endometriosis in both black and white women, although the mechanisms underlying this association remain to be clarified [28,29]. However, the present study is the first study of an Asian population to reveal an association between vitamin D deficiency and gynecological disease. Although bacterial vaginosis, which has been reported to be associated with vitamin D deficiency among pregnant women in western populations [30], was not evaluated in the present study, future research should investigate the relationship between bacterial vaginosis and vitamin D deficiency in Asian populations.

The Endocrine Society recently recommended that pregnant women consume at least 1500–2000 IU of vitamin D per day [31]. A recent randomized controlled trial showed that vitamin D supplementation for pregnant women of 4000 IU/day was both safe and the most effective level [32]. However, vitamin D supplementation is not part of most routine antenatal care programs in Korea. Although obstetricians in Korea usually recommend that pregnant women take a vitamin supplement during pregnancy, no consensus has yet been reached among physicians regarding whether the consumption of vitamin D-fortified food or specific vitamin D supplementation should be recommended. This lack of consensus is due at least in part to the lack of sufficient data on vitamin D status, vitamin D supplementation, and their associations with pregnancy-related outcomes to establish guidelines for the Korean population. The present study provides a foundation on which future research on vitamin D status and its associations with pregnancy-related outcomes in Korea can build. Vitamin D supplementation should only be recommended when many factors are taken into consideration. First, the designs and settings of the studies that inform these recommendations should be carefully considered. For instance, the current study included low-risk pregnant women and only looked at a few outcomes; moreover, the current study only enrolled participants with low vitamin D concentrations. Vitamin D expenditure should also be considered in the context of the plasma half-life of vitamin D. The details of supplementation regimens could also be important factors since different doses, boluses, and forms of supplementation could lead to varying biological effects. Moreover, geographical characteristics should also be considered because vitamin D needs can vary significantly within a country, particularly in countries that span large latitudes. During pregnancy, alterations in metabolism such as changes in vitamin D and calcium equilibrium compared with the non-pregnant state support the need for assessing vitamin D status and supplementation in the context of pregnancy. Improved assay methodologies that can detect vitamin D metabolites would also be useful for informing vitamin D supplementation needs, since most studies only report a minority of vitamin D metabolites. All these parameters should be taken into consideration in the design of future vitamin D supplementation trials.

The potential impact of vitamin D deficiency during pregnancy on maternal and neonatal health has attracted much interest in recent years. However, a causal link between vitamin D deficiency during pregnancy and adverse pregnancy-related outcomes remains to be determined using Hill’s criteria [33], which may be due in part to our limited knowledge. Although one report supported a possible link between a low 25(OH)D status and poor neonatal outcomes [5], the precise mechanisms underlying this association are yet to be determined. A recent systematic review and meta-analysis found that spontaneous preterm birth and childbirth with SGA were significantly associated with 25(OH)D levels <20 ng/mL [34]. In the present study, two pregnancy outcomes (preterm delivery and childbirth with SGA) were examined, and no significant association was found between vitamin D deficiency and either pregnancy outcome. These results are comparable with those of a study of pregnant Spanish women [3]. This agreement may be due to the small numbers of adverse pregnancy outcomes in both studies and the high prevalences of vitamin D deficiency among pregnant Korean women in the groups with and without adverse pregnancy-related outcomes. However, this finding limits the comparisons that can be made, thus warranting further research in this area. Another limitation of the current study is its lack of data about UVB levels and vitamin D intake. However, the current study is also valuable because it is the first to assess potential associations between vitamin D deficiency, preterm delivery, and SGA in a temperate climate region in an ethnically homogeneous Korean population.

In conclusion, our data indicate a high prevalence of vitamin D deficiency among pregnant women in Korea. Even during the summer months, a majority of pregnant women suffered from vitamin D deficiency. Being in the first trimester of pregnancy and the winter season were both associated with an increased risk of vitamin D deficiency in pregnant Korean women. Although no significant associations between vitamin D deficiency and preterm delivery or delivery of SGA babies were observed in the present study, this work will serve as a foundation for future research on vitamin D status and/or supplementation associated with pregnancy-related outcomes among pregnant Korean women.
